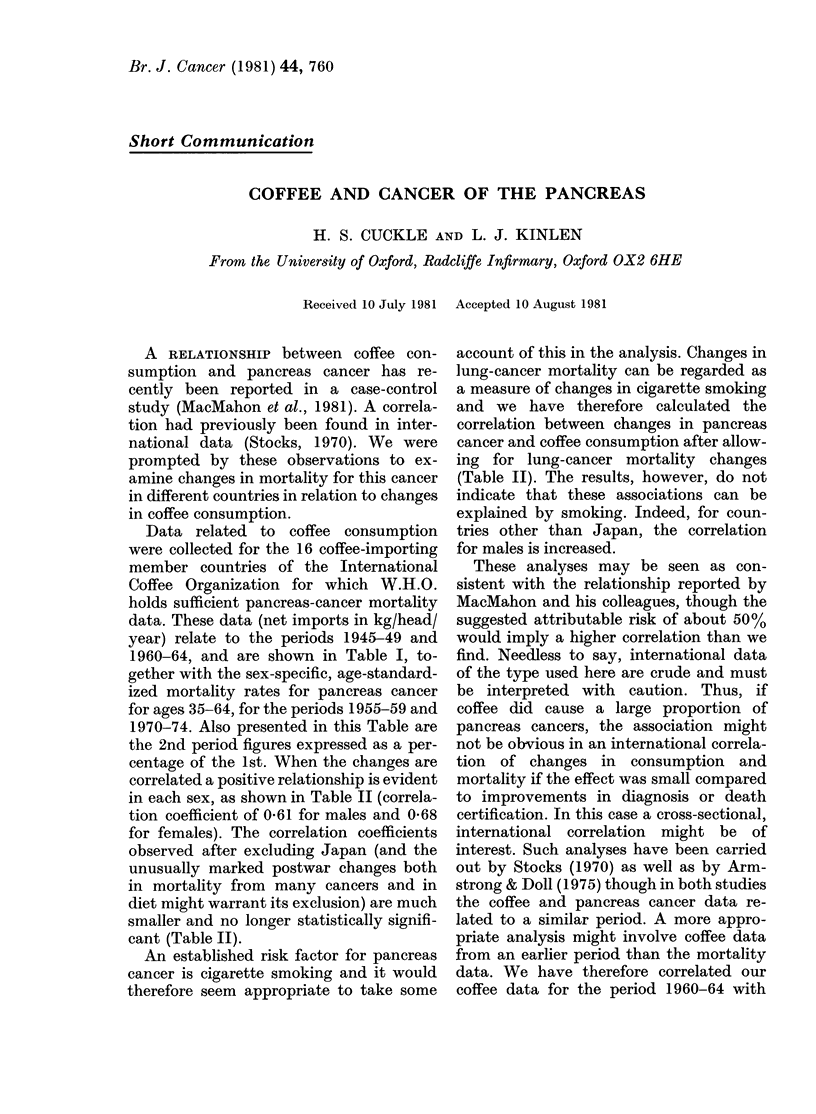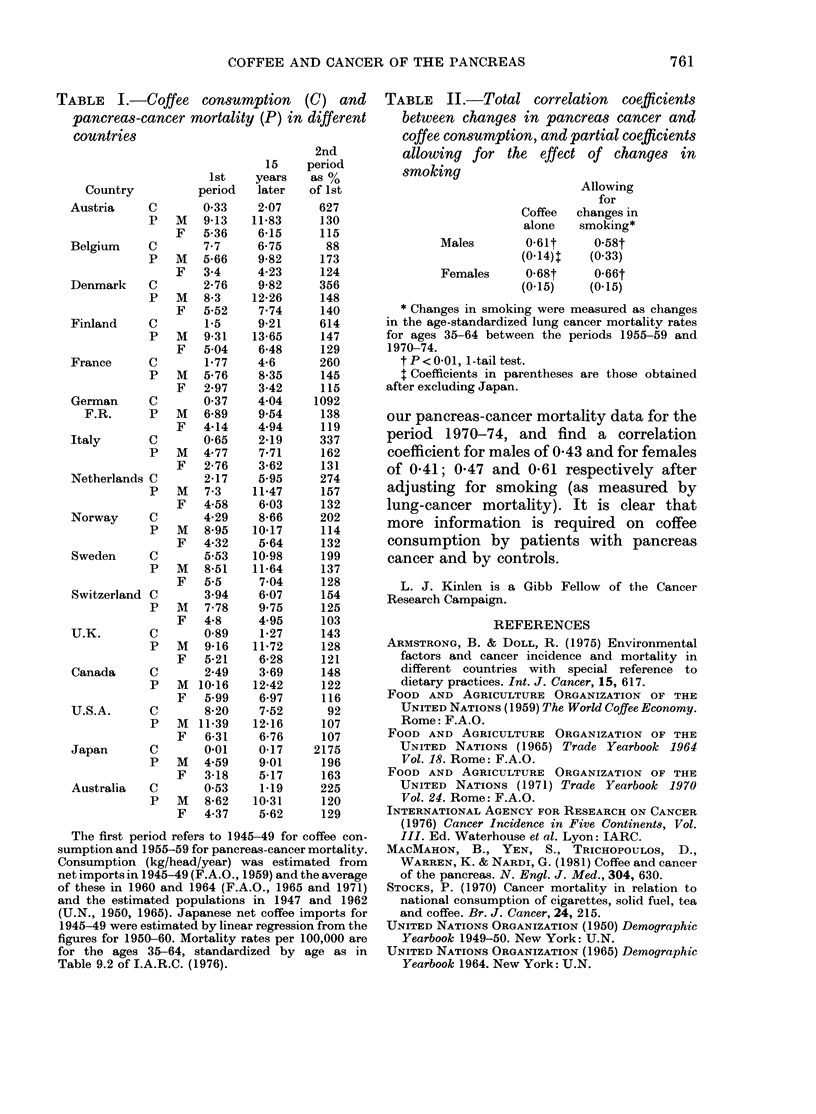# Coffee and cancer of the pancreas.

**DOI:** 10.1038/bjc.1981.266

**Published:** 1981-11

**Authors:** H. S. Cuckle, L. J. Kinlen


*Br. J. Cancer* (1981) **44**, 760

***Short Communication***

### COFFEE AND CANCER OF THE PANCREAS

H. S. CUCKLE AND L. J. KINLEN

*From the University of Oxford, Radcliffe Infirmary, Oxford OX2 6HE*

Received 10 July 1981 Accepted 10 August 1981

A RELATIONSHIP between coffee consumption and pancreas cancer has recently been reported in a case-control study (MacMahon *et al.*, 1981). A correlation had previously been found in international data (Stocks, 1970). We were prompted by these observations to examine changes in mortality for this cancer in different countries in relation to changes in coffee consumption.

Data related to coffee consumption were collected for the 16 coffee-importing member countries of the International Coffee Organization for which W.H.O. holds sufficient pancreas-cancer mortality data. These data (net imports in kg/head/year) relate to the periods 1945–49 and 1960–64, and are shown in Table I, together with the sex-specific, age-standardized mortality rates for pancreas cancer for ages 35–64, for the periods 1955–59 and 1970–74. Also presented in this Table are the 2nd period figures expressed as a percentage of the 1st. When the changes are correlated a positive relationship is evident in each sex, as shown in Table II (correlation coefficient of 0·61 for males and 0·68 for females). The correlation coefficients observed after excluding Japan (and the unusually marked postwar changes both in mortality from many cancers and in diet might warrant its exclusion) are much smaller and no longer statistically significant (Table II).

An established risk factor for pancreas cancer is cigarette smoking and it would therefore seem appropriate to take some account of this in the analysis. Changes in lung-cancer mortality can be regarded as a measure of changes in cigarette smoking and we have therefore calculated the correlation between changes in pancreas cancer and coffee consumption after allowing for lung-cancer mortality changes (Table II). The results, however, do not indicate that these associations can be explained by smoking. Indeed, for countries other than Japan, the correlation for males is increased.

These analyses may be seen as consistent with the relationship reported by MacMahon and his colleagues, though the suggested attributable risk of about 50% would imply a higher correlation than we find. Needless to say, international data of the type used here are crude and must be interpreted with caution. Thus, if coffee did cause a large proportion of pancreas cancers, the association might not be obvious in an international correlation of changes in consumption and mortality if the effect was small compared to improvements in diagnosis or death certification. In this case a cross-sectional, international correlation might be of interest. Such analyses have been carried out by Stocks (1970) as well as by Armstrong & Doll (1975) though in both studies the coffee and pancreas cancer data related to a similar period. A more appropriate analysis might involve coffee data from an earlier period than the mortality data. We have therefore correlated our coffee data for the period 1960–64 with

TABLE I.—*Coffee consumption (C) and pancreas-cancer mortality (P) in different countries*

| Country | | | 1st period | 15 years later | 2nd period as % of 1st |
|---|---|---|---|---|---|
| Austria | C | | 0·33 | 2·07 | 627 |
| | P | M | 9·13 | 11·83 | 130 |
| | | F | 5·36 | 6·15 | 115 |
| Belgium | C | | 7·7 | 6·75 | 88 |
| | P | M | 5·66 | 9·82 | 173 |
| | | F | 3·4 | 4·23 | 124 |
| Denmark | C | | 2·76 | 9·82 | 356 |
| | P | M | 8·3 | 12·26 | 148 |
| | | F | 5·52 | 7·74 | 140 |
| Finland | C | | 1·5 | 9·21 | 614 |
| | P | M | 9·31 | 13·65 | 147 |
| | | F | 5·04 | 6·48 | 129 |
| France | C | | 1·77 | 4·6 | 260 |
| | P | M | 5·76 | 8·35 | 145 |
| | | F | 2·97 | 3·42 | 115 |
| German | C | | 0·37 | 4·04 | 1092 |
| F.R. | P | M | 6·89 | 9·54 | 138 |
| | | F | 4·14 | 4·94 | 119 |
| Italy | C | | 0·65 | 2·19 | 337 |
| | P | M | 4·77 | 7·71 | 162 |
| | | F | 2·76 | 3·62 | 131 |
| Netherlands | C | | 2·17 | 5·95 | 274 |
| | P | M | 7·3 | 11·47 | 157 |
| | | F | 4·58 | 6·03 | 132 |
| Norway | C | | 4·29 | 8·66 | 202 |
| | P | M | 8·95 | 10·17 | 114 |
| | | F | 4·32 | 5·64 | 132 |
| Sweden | C | | 5·53 | 10·98 | 199 |
| | P | M | 8·51 | 11·64 | 137 |
| | | F | 5·5 | 7·04 | 128 |
| Switzerland | C | | 3·94 | 6·07 | 154 |
| | P | M | 7·78 | 9·75 | 125 |
| | | F | 4·8 | 4·95 | 103 |
| U.K. | C | | 0·89 | 1·27 | 143 |
| | P | M | 9·16 | 11·72 | 128 |
| | | F | 5·21 | 6·28 | 121 |
| Canada | C | | 2·49 | 3·69 | 148 |
| | P | M | 10·16 | 12·42 | 122 |
| | | F | 5·99 | 6·97 | 116 |
| U.S.A. | C | | 8·20 | 7·52 | 92 |
| | P | M | 11·39 | 12·16 | 107 |
| | | F | 6·31 | 6·76 | 107 |
| Japan | C | | 0·01 | 0·17 | 2175 |
| | P | M | 4·59 | 9·01 | 196 |
| | | F | 3·18 | 5·17 | 163 |
| Australia | C | | 0·53 | 1·19 | 225 |
| | P | M | 8·62 | 10·31 | 120 |
| | | F | 4·37 | 5·62 | 129 |

The first period refers to 1945–49 for coffee consumption and 1955–59 for pancreas-cancer mortality. Consumption (kg/head/year) was estimated from net imports in 1945–49 (F.A.O., 1959) and the average of these in 1960 and 1964 (F.A.O., 1965 and 1971) and the estimated populations in 1947 and 1962 (U.N., 1950, 1965). Japanese net coffee imports for 1945–49 were estimated by linear regression from the figures for 1950–60. Mortality rates per 100,000 are for the ages 35–64, standardized by age as in Table 9.2 of I.A.R.C. (1976).

TABLE II.—*Total correlation coefficients between changes in pancreas cancer and coffee consumption, and partial coefficients allowing for the effect of changes in smoking*

| | Coffee alone | Allowing for changes in smoking* |
|---|---|---|
| Males | 0·61† | 0·58† |
| | (0·14)‡ | (0·33) |
| Females | 0·68† | 0·66† |
| | (0·15) | (0·15) |

* Changes in smoking were measured as changes in the age-standardized lung cancer mortality rates for ages 35–64 between the periods 1955–59 and 1970–74.

† $P < 0{\cdot}01$, 1-tail test.

‡ Coefficients in parentheses are those obtained after excluding Japan.

our pancreas-cancer mortality data for the period 1970–74, and find a correlation coefficient for males of 0·43 and for females of 0·41; 0·47 and 0·61 respectively after adjusting for smoking (as measured by lung-cancer mortality). It is clear that more information is required on coffee consumption by patients with pancreas cancer and by controls.

L. J. Kinlen is a Gibb Fellow of the Cancer Research Campaign.

### REFERENCES

ARMSTRONG, B. & DOLL, R. (1975) Environmental factors and cancer incidence and mortality in different countries with special reference to dietary practices. *Int. J. Cancer*, **15**, 617.

FOOD AND AGRICULTURE ORGANIZATION OF THE UNITED NATIONS (1959) *The World Coffee Economy.* Rome: F.A.O.

FOOD AND AGRICULTURE ORGANIZATION OF THE UNITED NATIONS (1965) *Trade Yearbook 1964 Vol. 18.* Rome: F.A.O.

FOOD AND AGRICULTURE ORGANIZATION OF THE UNITED NATIONS (1971) *Trade Yearbook 1970 Vol. 24.* Rome: F.A.O.

INTERNATIONAL AGENCY FOR RESEARCH ON CANCER (1976) *Cancer Incidence in Five Continents, Vol. III.* Ed. Waterhouse *et al.* Lyon: IARC.

MACMAHON, B., YEN, S., TRICHOPOULOS, D., WARREN, K. & NARDI, G. (1981) Coffee and cancer of the pancreas. *N. Engl. J. Med.*, **304**, 630.

STOCKS, P. (1970) Cancer mortality in relation to national consumption of cigarettes, solid fuel, tea and coffee. *Br. J. Cancer*, **24**, 215.

UNITED NATIONS ORGANIZATION (1950) *Demographic Yearbook* 1949–50. New York: U.N.

UNITED NATIONS ORGANIZATION (1965) *Demographic Yearbook* 1964. New York: U.N.